# Bacterial Cellulose-Derived Sorbents for Cr (VI) Remediation: Adsorption, Elution, and Reuse

**DOI:** 10.3390/polym16182605

**Published:** 2024-09-14

**Authors:** Uriel Fernando Carreño Sayago, Vladimir Ballesteros Ballesteros, Angelica María Lozano Aguilar

**Affiliations:** Faculty of Engineering and Basic Sciences, Fundación Universitaria los Libertadores, Bogotá 111221, Colombia; vladimir.ballesteros@libertadores.edu.co (V.B.B.); amlozanoa@libertadores.edu.co (A.M.L.A.)

**Keywords:** cellulose bacterial, chromium, isotherm, kinetics

## Abstract

The search for adsorbents that are non-toxic and low cost with a high adsorption capacity and excellent recyclability is a priority to determine the way to reduce the serious environmental impacts caused by the discharge of effluents loaded with heavy metals. Bacterial cellulose (BC) biomass has functional groups such as hydroxyl and carbonyl groups that play a crucial role in making this cellulose so efficient at removing contaminants present in water through cation exchange. This research aims to develop an experimental process for the adsorption, elution, and reuse of bacterial cellulose biomass in treating water contaminated with Cr (VI). SEM images and the kinetics behavior were analyzed with pseudo-first- and pseudo-second-order models together with isothermal analysis after each elution and reuse process. The adsorption behavior was in excellent agreement with the Langmuir model along with its elution and reuse; the adsorption capacity was up to 225 mg/g, adding all the elution processes. This study presents a novel approach to the preparation of biomass capable of retaining Cr (VI) with an excellent adsorption capacity and high stability. This method eliminates the need for chemical agents, which would otherwise be difficult to implement due to their costs. The viability of this approach for the field of industrial wastewater treatment is demonstrated.

## 1. Introduction

Alternative and non-conventional processes for water treatment are the subject of investigation in research centers around the world. It is imperative that these processes are effective, affordable, and straightforward to develop and implement [1,2,3]. The lack of implementation of treatment systems in various industries is largely attributed to the high costs associated with such systems. The use of cellulolytic biomass represents a promising alternative method for the treatment of water contaminated with heavy metals [4,5,6,7,8]. It has the capacity to chemisorb these contaminants and remove them from industrial effluents, which are the primary source of significant environmental and social impacts. It is regrettable that heavy metals continue to represent a significant challenge to the quality of water sources in Latin America, particularly in chemical industries where the lack of adequate treatment systems is a prevalent issue [7,9]. Bacterial cellulose (BC) represents an environmentally friendly alternative due to the minimal impact of its production process, which is both economical and straightforward [10,11]; In contrast to plant biomass, which contains cellulose, hemicellulose, and lignin, BC is composed entirely of cellulose, which is significant in the context of heavy metal retention, as lignin can negatively affect this process [12,13,14].

Laboratory-scale experiments have shown that plant biomass can adsorb approximately 30 mg/g of heavy metals, as determined through kinetic and isotherm studies [15,16]. Bacterial cellulose is an amazing material with exceptional mechanical properties. Moreover, it is also biocompatible and biodegradable, which makes it perfect for biomedical applications and various environmental processes [17]. For example, it has been successfully used in industrial wastewater treatment [18]. In fact, it has demonstrated a maximum adsorption capacity of 69 mg/g for Cd (II) and 116 mg/g for crystal violet [19]. In chemical treatment research, this biomass has shown promising results in adsorbing Cr (VI), reaching 95 mg/g [20]. Bacterial cellulose biomass is a type of extracellular cellulose mainly produced in vitro by Acetobacter bacteria. It is based on glucose linked by β-1,4 glycosidic bonds. The BC microfibrils intertwine, forming a unique network structure with hydroxyl groups (OH) that are important in the chemisorption processes of heavy metals [21,22,23,24]. Biomass has a large amount of hydroxyl (OH) groups, which are mainly responsible for the cationic exchange between biomass hydrogen ions and heavy metals [25,26,27]. In addition, its unique network of ultrafine nanofibers interconnected in 3D can ensure the rapid transport of ions, increasing the contact area with contaminants to improve adsorption capacity [28,29]. The application of kinetic models, in particular the pseudo-second-order model, enables the description of the complexation between the biomass and the contaminant. This, in turn, allows the determination of characteristic parameters that can be employed to model and scale the treatment system [25,30].

In general, the ability to be recycled and reused is a desirable quality in an ideal adsorbent. As a result, this is a crucial design parameter when evaluating biomass in the process of developing alternative treatment systems. It is essential to assess the behavior of the adsorbent materials during elution processes. It has been demonstrated that bacterial cellulose biomass exhibits satisfactory responsiveness to elution processes and subsequent recycling, with the capacity to withstand up to five additional treatment cycles [31]. These processes are designed to facilitate the recycling of bacterial cellulose biomass and enhance the efficiency of water treatment. A chemical agent that is frequently employed in elution processes is EDTA, which possesses a notable ability to elute adsorbent materials [32].

The aim of this research is to develop an experimental process for the adsorption, elution, and reuse of bacterial cellulose biomass in the treatment of water contaminated with Cr (VI). The biomass has been characterized by FTIR and SEM images, and the adsorption mechanisms of first- and second-order models have been analyzed together with isothermal analysis after each elution and reuse process, with the intention of obtaining critical parameters for decision-making in order to develop prototypes on a larger scale.

## 2. Methods and Materials

### 2.1. Bacterial Cellulose Production

**Figure 1:**
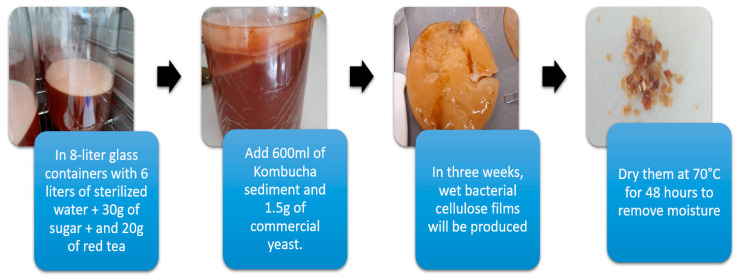
Production of bacterial cellulose.

Bacterial cellulose (BC) films were produced using a tea and sugar culture medium obtained from the bioprocess laboratory of the Universidad Fundación Los Libertadores. The culture medium was prepared with 3 g of sugar and 5 g of commercial yeast dissolved in distilled water and heated to (80 °C). After cooling, 300 mL of Kombucha sediment was added and taken to a 15 L laboratory incubator (IO Xtemp Series Dual Incubator Oven, Hettich, Föhrenstr, Germany). with a temperature of 37 °C. pH samples were taken on site for an internal control, which had a neutral pH. After three weeks, the bacterial cellulose biomass production was dried at 70 °C for 48 h to remove moisture (see Figure 1). The bacterial cellulose was ground until it has a diameter of 0.212 mm. This process allowed the production of 70 g of bacterial cellulose in two weeks, using the method based on [7,9].

### 2.2. Batch Adsorption

Experiments were conducted in a 100 mL glass vessel with constant agitation (IKA Ks 4000 shaker, Hettich, Föhrenstr Germany) at 20 °C and 150 rpm. The data were recorded at 20 min intervals until 180 min had elapsed. The sample size was 20 µm, which was subsequently transferred to the centrifuge (KASAI MIKRO 200, Hettich, Föhrenstr Germany). The research parameters included initial chromium concentrations of 50, 100, 200, 300, and 600 mg/L. At each time interval, samples were taken. At 20 min intervals, the residual chromium concentration was analyzed. All experiments were conducted in triplicate, and the final values were averaged.

In the present investigation, the tests were performed under neutral pH conditions of the samples, which favors the adsorption process in this type of biomass [32].

The adsorption capacity was determined by suspending 0.3 g of biomass in 100 mL of a Cr (VI) solution for 140 min at 200 rpm, with samples taken every 20 min prior to the determination of the residue. The sediment was then discarded. All procedures were conducted in duplicate.

Aliquots of the reaction mixture were subjected to analysis in order to ascertain the residual concentration of chromium, using a UV–Vis spectrophotometer (UV84 Hettich, Föhrenstr Germany). The measurement uncertainty of the study indicates that measurements of heavy elements, specifically Cr (VI), can be performed with an uncertainty level of approximately 3.95%.

The quantity of Cr (VI) residue was calculated using the diphenylcarbazide method. The phosphate buffer solution was prepared by adjusting the pH to 2 with 90% H_3_PO_4_. Into an Eppendorf tube, 200 µL of 0.5% diphenylcarbazide (in acetone), 900 µL of phosphate buffer, and 100 µL of residual sample were added. The absorbance was measured at 540 nm, following transfer from an adsorption cell.

A spectrophotometer (Evolution 300 spectrophotometer) was used to monitor changes in absorbance. All procedures for chromium determination, for water and substrates, were performed implementing the APHA (American Public Health Association Procedure) for standard testing (standard methods for the examination of water and wastewater) [15].

### 2.3. The Desorption–Adsorption

Following the completion of the Cr (VI) adsorption process, the chromium-laden biomass was subjected to an elution process and subsequent recycling. This involved the washing of the biomass with distilled water and its transfer to an Erlenmeyer flask containing 20 mL of EDTA (1 g/L) at 25 °C for a period of six hours with constant stirring.

The bacterial cellulose biomass was then washed with distilled water and left to dry, allowing it to be reused as many times as necessary for the purposes of this investigation. The tests were conducted under neutral pH conditions. The aforementioned elution and reuse were based on the methodology outlined in Reference [32].

Analysis of treatment

BC (0): Biomass cellulose without elution;
BC (1): Biomass cellulose Elution 1;
BC (2): Biomass cellulose Elution 2;
BC (3): Biomass cellulose Elution 3.

All experiments were performed with the same initial concentration parameters to determine the isotherms, which were initial chromium concentrations of 50, 100, 200, 300, and 600 mg/L.

### 2.4. Adsorption Models

Table 1 shows the summary of the models of the isotherm and kinetics.

### 2.5. FTIR

The materials were characterized by Fourier transform infrared spectroscopy (79 Jasco FTIR 430, Tokyo, Japan) to measure the IR spectra in a spectral range of 4000–400 cm^−1^, with a resolution of 4 cm^−1^ and a scanning speed of 2 mm s^−1^.

### 2.6. SEM and EDS Analysis

The results observed in investigations involving cellulose and heavy metals have been confirmed by SEM and EDS analysis using the TESCAN FE-MEB LYRA3 Focused Ion Beam Scanning Electron Microscope (Brno, Czech Republic). The MEB features an integrated microanalysis system for Energy Dispersive X-ray Spectroscopy (EDS).

### 2.7. Measurement of the Pore Volume of Bacterial Cellulose

The density of the total biomass used in the experiment has a weight of 0.3 g, occupying different volume spaces, and is calculated with the following equation:

(7) $$p(Cb)=\frac{m(Cb)}{V(Cb)}$$

where V(Cb) is the volume occupied by the biomass. The total volume of biomass in the batch experiment (including spaces between pores and air) is a fundamental parameter. The simplest particle will have a direct relationship with the contaminant, and the biomass will have an indirect relationship with the contaminant. The more space there is between particles, the better it will be for the treatment. To obtain the density of the microparticle, Equation (8) is used.

(8) $$\rho p=\frac{mp}{Vp}$$

The mass of the microparticle (m_p_) is its weight and the volume of the microparticle (v_p_) is obtained by the following equation:

(9) $$Vp=\frac{4\pi r^{3}}{3\;}$$

(10) $$\varepsilon =1-\frac{p(Cb)}{pp}$$

In this research, the radius of the tiny particle (r) will be treated as a dependent variable. Its diameter will be obtained in meshes and classified by size. This is the relationship between the densities of the particle and the density of the biomass occupied in the experiment [33]. This equation will be fundamental due to the relationship between the general biomass and its small particles.

## 3. Result

**Figure 2:**
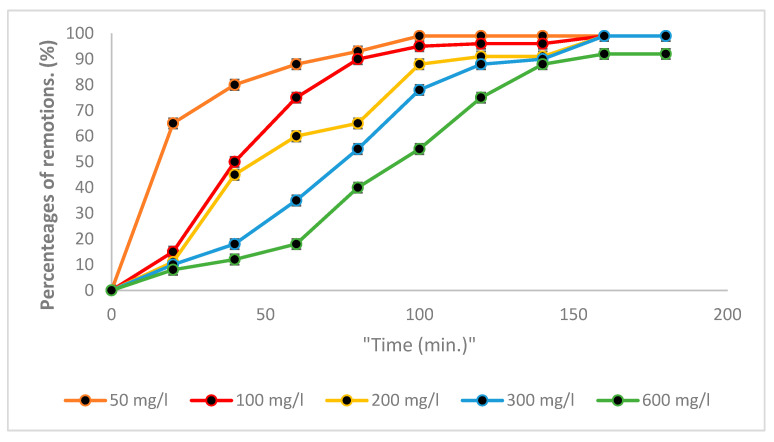
Percentages of Cr (VI) removal with BC.

Figure 2 shows the removal percentages of Cr (VI) from BC.

The Cr (VI) removal with BC was significant within the first 50 min, for the initial concentrations of 50 mg/L, at which more than 80% of Cr (VI) was removed, with efficiencies reaching 95% at the end of the process. For 100 and 200 mg/L, around 45% removal was obtained in the first few minutes, with removals of 95% subsequently being reached. The concentration of 300 mg/L removed more than 50% within the first few minutes, and finally reached a removal efficiency of 90%. Similarly, the initial concentration of 600 mg/L removed less than 30% during the initial process, and finally a removal efficiency of 88% was achieved for Cr (VI), indicating that this biomass is a potent bioremediation effector of Cr (VI) present in water, whereas when the initial concentration of this heavy metal increases it could also be affected due to the possible saturation of active sites [7,9].

### 3.1. Adsorption Mechanism by Bacterial Cellulose Biomass

**Figure 3:**
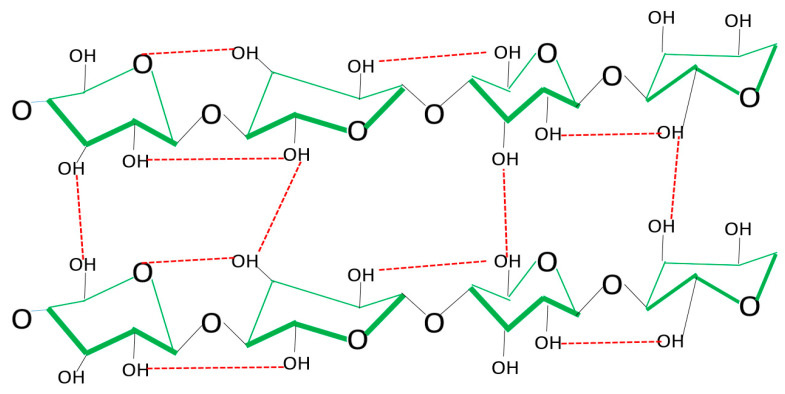
Biochemical structure of bacterial cellulose.

In bacterial cellulose biomass, there are hydroxyl (OH) groups where the various heavy metal Cr (VI) ions would be accommodated. The difference with plant cellulose is the absence of lignin in this polymer, which provides many more of these groups to ensure better removal processes for these contaminants. Figure 3 shows BC with β-1,4 glycosidic covalent bonds. Hydroxyl groups are abundant in each glucose linkage on carbons C_2_, C_3_, and C_6._ The special features of bacterial cellulose are shown in Figure 3.

**Figure 4:**
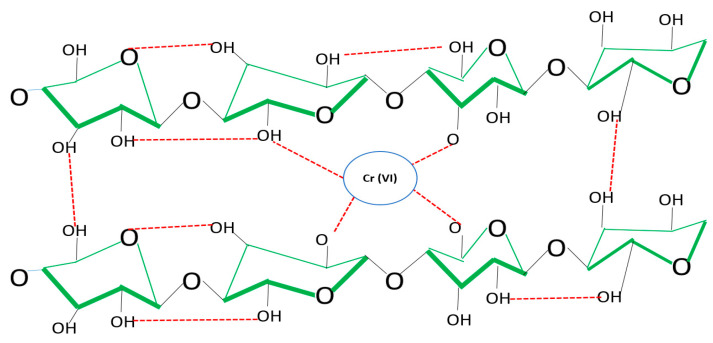
Cr (VI) adsorption process.

Various glucose linkages, reinforced by hydrogen bonds (red band), can be observed both within and between glucose chains. Subsequently, Cr (VI) is chemically adsorbed by the continuous interaction between hydroxyl groups and hydrogen ions (red band), resulting in retention in the biomass as shown in Figure 4 [34,35].

Figure 4 shows that eight glucose molecules with a C_6_H_10_O_5_ structure are adsorbing two chromium atoms, which are part of the structural branching of cellulose. The structural complexity in the adsorption of this heavy metal and the potential of EDTA make the elution process and subsequent reuse of this biomass efficient without seriously affecting the latter. Being 100% cellulose, its biochemical structure makes it simpler than other cellulose, and this parameter is essential in the elution and reuse processes [36].

### 3.2. FTIR

**Figure 5:**
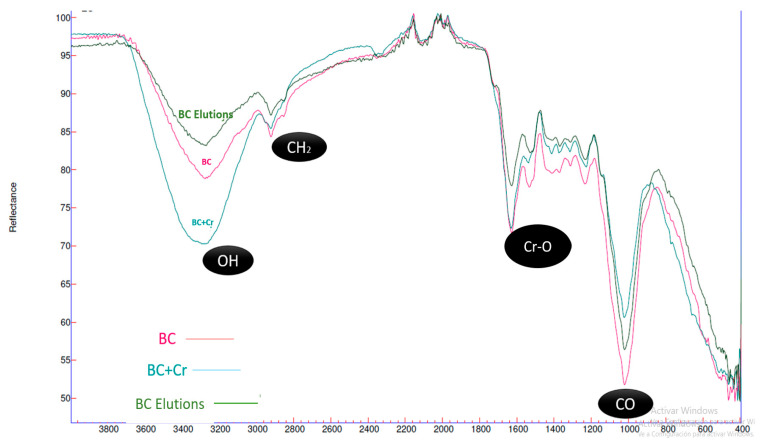
Characteristic spectra of BC before and after Cr (VI) adsorption.

Figure 5 shows the characteristic spectra of BC before and after Cr (VI) adsorption. The hydroxyl groups (OH) are visible in the 3400 bands, along with the characteristic band of bacterial cellulose.

Figure 5 illustrates the analysis of the chemical structures of bacterial cellulose by FTIR. The absorption peaks observed at 3400 cm^−1^ are attributed to OH stretching vibrations, which represent the primary chemical structures involved in the chemisorption process during water treatment [36]. These are the active sites where cation exchange occurs, and the alterations in this peak are crucial due to the interaction between the contaminant and the hydroxyl group [16]. Following the adsorption process between biomass and heavy metal Cr (VI), it was observed that the peak at 3400 cm^−1^ was responsible for the adsorption of Cr (VI); this is due to the cation exchange between hydrogen ions of the biomass and contaminant Cr (VI) [20]. This is a characteristic process of cellulose biomass adsorption in heavy metal treatment [37]. A vibrational expansion can also be observed at the 1632 cm^−1^ peak, due to the incorporation of the amide group (NH), a component of EDTA. In the subsequent elution processes, a decrease in the vibrational expansion can be identified at the absorption peak of 2920 cm^−1^, corresponding to the stretching of CH_2_, and at the peak of 1000 cm^−1^ corresponding to CO; this is due to a possible wear of the biochemical structure of bacterial cellulose caused by EDTA [32]. After undergoing a chemical elution process using EDTA, the eluted BC biomass indicates a decrease in vibrational expansion at the 1600 cm^−1^ peak bands corresponding to Cr-O, demonstrating the ability of this chemical agent to elute bacterial biomass.

### 3.3. Measurement of the Pore Volume of Bacterial Cellulose

The particle diameter was approximately 0.0212 mm, which demonstrated favorable outcomes in relation to the correlation between the biomass and the contaminant. Table 2 presents the final results of the analysis of the relationship between densities, and the table also demonstrates that the biomass was identical in all experiments.

Equations (7)–(10) were used to determine the relationships between the densities (ε) of the bacterial cellulose biomass used in the batch experiment, in which 0.3 g of it was used, remaining constant in the subsequent elution and reuse, and the density of the microparticle. The relationship between these densities is a design parameter used in this type of treatment, where this value effectively correlates the active sites of the biomass with Cr (VI), and the subsequent elutions also demonstrated a favorable correlation, as evidenced by all the biomass results, which remained within the desired ranges of (ε), where it is established that this value should be between 0.5 and 0.8. A compact relationship between both densities favors the adsorption process [38,39,40]. It can be observed that the volumes increased after the elution process with EDTA, where traces of this reagent were impregnated, thus increasing its volume, both in the microparticle and in the volume occupied by the biomass. The optimal relationship was observed with the BC (0) biomass, with an index of 0.68.

### 3.4. Isotherms

**Figure 6:**
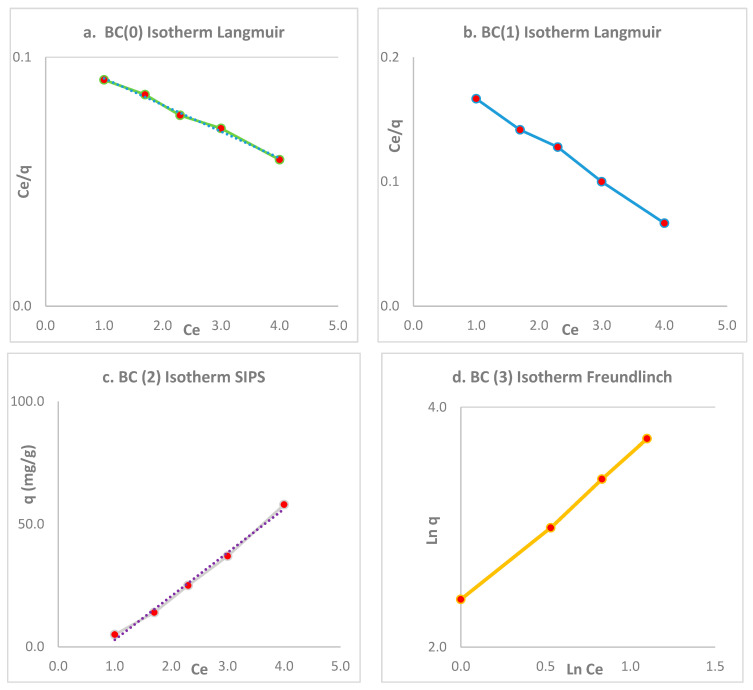
Relationship between adsorption capacities and equilibrium concentrations and corresponding adjustments. (**a**) BC (0) with the Langmuir isotherm, (**b**) BC (1) with the Langmuir isotherm; (**c**) BC (2) with the SIPS isotherm, and (**d**) BC (3) with the Freundlich isotherm.

The figure shows the relationship of bacterial cellulose, between the adsorption capacities (q_e_) and the different equilibrium concentrations (C_e_) obtained in each of the initial concentrations of 600, 300, 200, 100, and 50 mg/L of Cr (VI). Through Equation (4), the adsorption capacities of each bacterial cellulose biomass can be established. Like in Figure 6, all biomass eluted with EDTA was subjected to the reuse process under the same conditions until the cellulose no longer carried out significant removal.

By increasing the initial concentration to 600 mg/L, the removal efficiency gradually decreased; this fact indicated that Cr (VI) ions fill the active sites (OH) of the bacterial cellulose biomass. However, the adsorption capacity in equilibrium has a correlation with the initial concentration; as the initial concentrations increase, the capacity tends to increase.

Cr (VI) ions fill the active sites (OH) in the bacterial cellulose biomass; a characteristic of this biomass is its homogeneity in the adsorption processes, with a maximum adsorption capacity at 75 mg/g, which in this condition fits a Langmuir isotherm, considering that this isotherm assumes that all the active sites on the surface are energetically homogeneous. The Langmuir equation is based on the following considerations: (1) there is a constant number of accessible sites on the surface of the adsorbent, and all of them are energetically equivalent and independent; (2) when a contaminant is adsorbed on a site, no more contaminants can be adsorbed on that site; (3) the adsorption is reversible; and (4) there is no interaction between contaminant molecules that occupy neighboring sites [19].

A representative fit of 0.99 R^2^ can be observed in Figure 6.

Subsequently, experimental processes were carried out to determine if the elution and reuse processes using EDTA affect the adjustment behavior to this isotherm. After the first process of chemical elution and biomass reuse under the same conditions, a fit to the Langmuir isotherm in BC (1) was evident, but there was also a significant fit to the SIPS isotherm. The Langmuir parameter $K_{l}$ indicates that the adsorption bond energy between the BC biomass and Cr (VI) is high at 0.03; this value reflects a stronger bond energy, which will lead to a higher adsorption capacity [41,42].

The Langmuir model has been shown to have a high goodness of fit for all models, particularly for the uneluted bacterial cellulose biomass. The adsorption capacity evaluations derived from this model could be employed in future treatment system designs. Furthermore, it has been established that the bacterial cellulose biomass constitutes a homogeneous monolayer coating, and that the Langmuir isotherm is the isotherm that best describes this process [43]. Table 3 shows the representative isotherms.

The SIPS and Freundlich isotherms are more appropriate after the elution processes to represent the bacterial cellulose biomass. The SIPS model represented BC (2), as a route of transition from the Langmuir model to the Freundlich model, between the processes of elution and reuse of the BC biomass. The SIPS model is an empirical model like the Freundlich model, and in its mathematical form it is similar to the Langmuir model; however, this model represents a finite limit when the concentration of the contaminant in the aqueous phase is high enough, and this model is also known as the Langmuir–Freundlich model. Due to this, M_s_ represents the heterogeneity of the adsorption surface [44]. BC (3) was fitted to the Freundlich isotherm; this was due to the transformation after elution of the biomass, without affecting the functional groups and therefore reducing its capacity, since the electrostatic attractions between the metal ions and the biomass were not affected, and this is indicated by this isotherm [45].

The Freundlich adsorption isotherm model is used to model non-ideal and reversible adsorption processes. Unlike the Langmuir model, this model is not restricted to the formation of monolayers, and its use for multilayer adsorption is feasible when there is a non-uniform distribution of the adsorption heat and affinities along the heterogeneous surface. The linearity range at low contaminant levels and the saturation effects at high contaminant levels are not explained by this isotherm. Therefore, it can be concluded that the Freundlich isotherm cannot describe the saturation behavior of an adsorbent [46].

The adsorption capacity of biomass increased to 225 mg/g after up to four cycles of elution and continuous reuse. This value represents the sum of all adsorption capacities. This parameter is crucial in scientific research processes, particularly in the development of a pilot-scale prototype. Pineapple peels exhibited high capacities after elution processes, reaching capacities of 66 mg/g [47]. Experiments with the sum of Cd (II) capacities showed a biomass absorbance of 84 mg/g from Citrus maxima peel [48]. In the adsorption capacity processes with Cr (VI), lignocellulosic biomass and EDTA reached 156 mg/g [49].

### 3.5. Kinetic Studies

In all of the aforementioned models, the nonlinear form of the equations was employed in order to identify the most appropriate kinetic model that would best represent the adsorption processes. They are commonly used to predict the adsorption rate and provide crucial information for designing and modeling the treatment process [50]. The high adsorption capacity depends on the availability of the active sites, resulting in rapid adsorption in the first few minutes and reaching equilibrium in less than two hours [51].

The experimental values for all biomasses before and after the elution processes align with the values of adsorption capacities calculated from the pseudo-second-order model, further reinforcing the reliability of this model [52]. The adsorption outcomes demonstrate that the pseudo-second-order kinetics were driven by chemisorption, involving valence forces or cationic exchange between the electrons of the active sites of the biomass and Cr (VI) [53,54,55,56].

**Figure 7:**
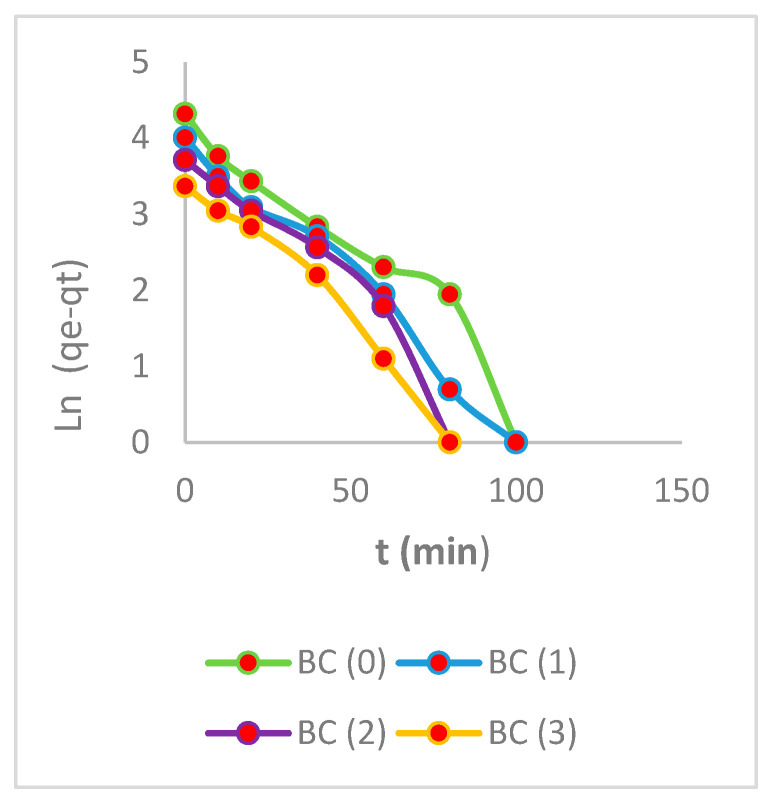
Pseudo-first order.

**Figure 8:**
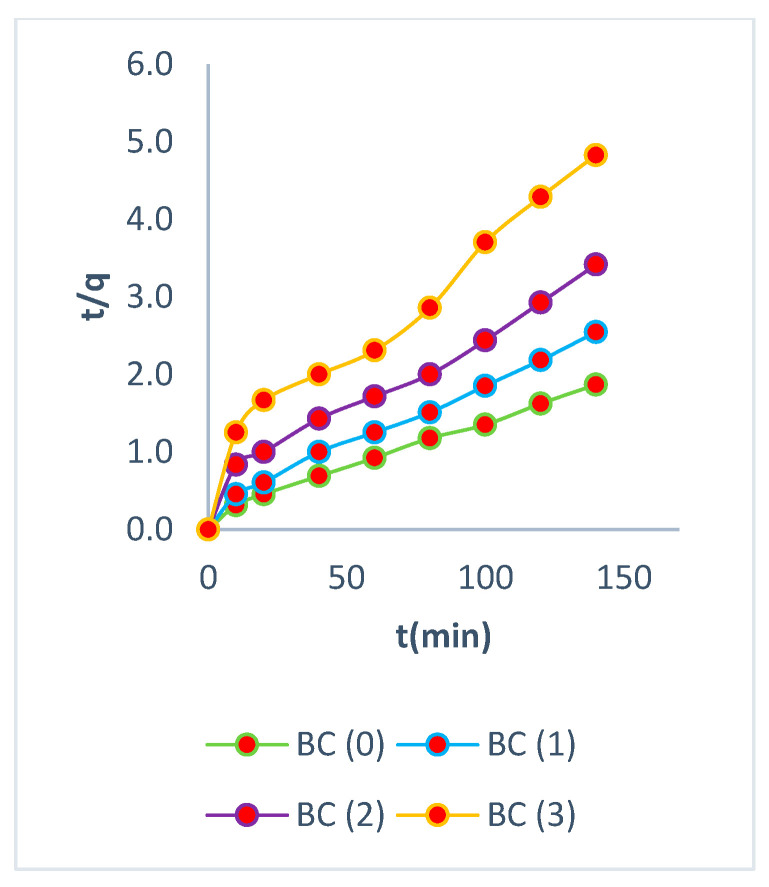
Pseudo-second order.

**Figure 9:**
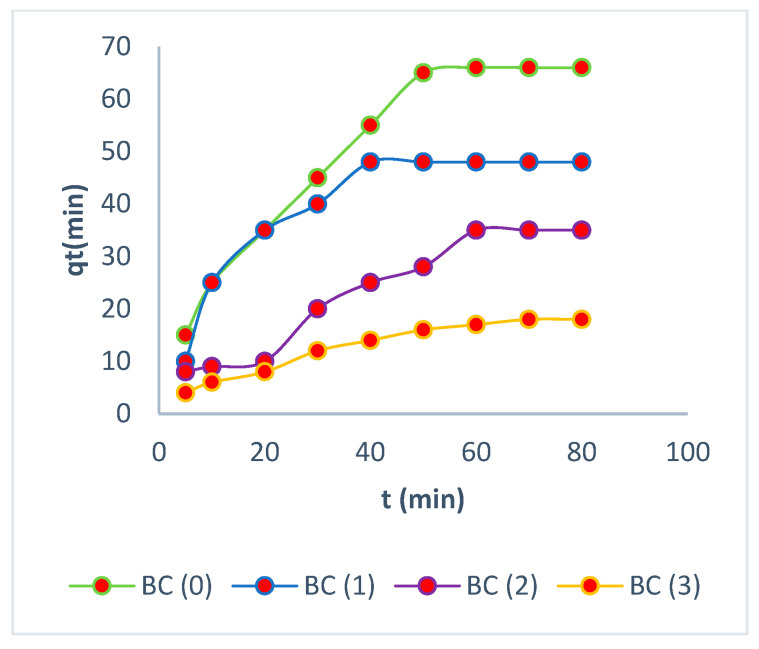
Intraparticle diffusion.

Prior to the elution processes, the experiment was found to fit the second-order model, as determined by Equations (5)–(7). The equilibrium adsorption capacity was determined to be 76 mg/g, with higher values in R^2^. Figure 7, Figure 8 and Figure 9 show a high adsorption capacity in the processes prior to elution.

BC showed a strong correlation with a second-order model. This is due to the rapid exchange of Cr (VI) ions with hydrogen ions present in the biomass. BC biomass has a large number of active sites, allowing a high-rate exchange rate of 1.4 × 10^−3^ (g/mg × min), where the bacterial cellulose layers appeared homogeneous in the isotherms, explaining their high adsorption capacities. The parameters are shown on Table 4.

Similar results were obtained with amide-functionalized cellulose biomass [57]. The BC (1) biomass also fitted a second-order model, with a lower but still significant adsorption rate of 1.5 × 10^−3^ (g/mg × min), similar to BC (0) biomass. This demonstrates the rapid rate of bacterial cellulose biomass after the first elution with EDTA. Furthermore, the BC (2) biomass has a rate constant of 1.16 × 10^−3^ (g/mg × min), further highlighting the resilience of this biomass after elution and reuse. BC (3) was fitted to the first-order model, achieving rate values of 1.18 × 10^−3^ (g/mg × min). Previous research has demonstrated rates of less than 1 × 10^−3^ (g/mg × min) without elution processes [58]. The first- and second-order models were employed to represent the adsorption kinetics model of Cr (VI) in the various biomasses, together with the representative values of the fit, as presented in Table 4. It was observed that all the biomasses exhibited a fit that was better represented by the second-order model, with the exception of the biomass BC (3), which demonstrated a fit that was more closely aligned with the first-order model. These findings elucidate the underlying surface processes that involve both chemisorption and physisorption in the adsorption of Cr (VI) on bacterial cellulose biomasses [59].

**Figure 10:**
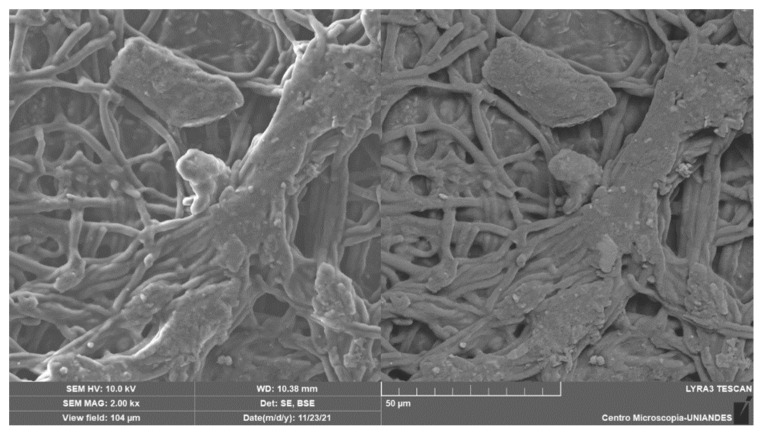
Photomicrograph SEM of bacterial cellulose (BC).

This biomass can retain up to 246 mg/g Cr (VI) after multiple elutions and reuses, making it one of the most effective biomasses for adsorption. It is noteworthy that this bacterial cellulose biomass is inexpensive and does not require any chemical processing. Initially, it conforms to an external layer model, but during the elution processes, it adjusts to an intraparticle process due to the loss of adsorption capacity caused by biomass deterioration and the loss of active sites where the biomass had been protonated due to different EDTA elutions. Figure 10 shows the adjustment to the intraparticle model, achieving balance.

The photomicrograph in Figure 10 shows BC analyzed through SEM. In order to determine the physicochemical characteristics of the adsorption processes, we characterized each elution process and identified the potential effects of this biomass.

**Figure 11:**
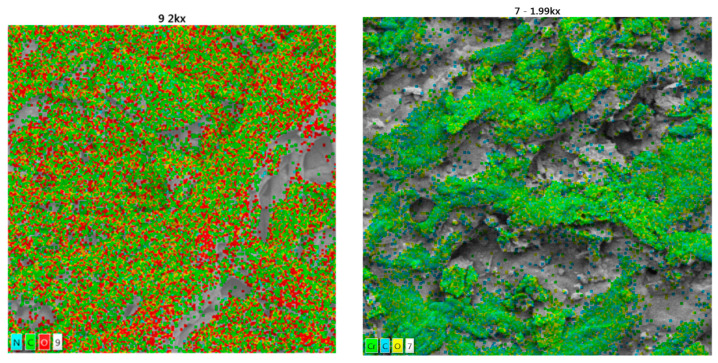
Photomicrographs of BC with the location of each representative element.

Figure 10 displays a fibrillar structure that appears homogenous, with fine diameters in the nanometer range. Additionally, there are aggregates of larger cellulose fibrils present. In chemical adsorption processes, improved removal performance can be achieved when heavy metal particles and microfibrils have a closer relationship, reaching nanometers; this is because of the direct relationship between the biomass and the contaminant [60], which facilitates the cation exchange process. Therefore, this biomass is effective in removing these contaminants [61]. The photomicrographs depict bacterial cellulose, showing the location of each representative element before and after the Cr (VI) adsorption process in Figure 11.

BC can be observed with the location of each representative element, where after the adsorption process the predominance of Cr (VI) can be seen, evidencing chemisorption coupled with accommodation in the sample, complementing Table 4 with a chemisorbed percentage of 13.3% of this heavy metal. The regularity of the complexations formed between the biomass and Cr (VI) during biosorption suggests that the reaction occurred on the material’s surface, which was well distributed. This observation is similar to that reported in the literature [62,63]. Characterizations after chemical elution processes through EDTA are also shown in Table 5.

**Figure 12:**
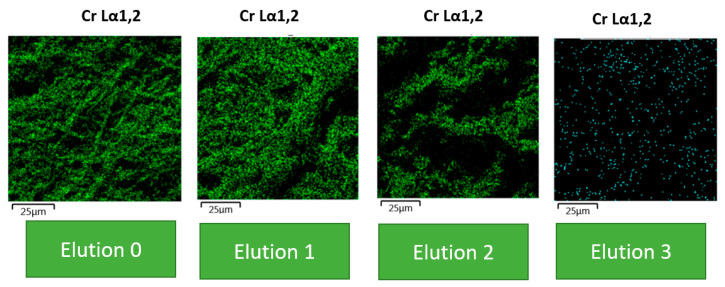
Photomicrographs of BC in each elution process.

For the complete analysis, semi images were carried out after each elution and reuse process, as seen in Figure 12.

After the elution processes (Figure 12), the reuse of biosorption suggests the relationship occurred in certain non-homogeneous sites. The SEM images show the amounts of Cr (VI) in green, and these values are summarized in Table 4 as percentages. In Elution 0, the biomass remained unaffected and retained its entire biochemical structure, with 13.5% of Cr (VI) chemisorbed. However, as the elution processes with EDTA and subsequent reuse were carried out, the biomass began to show signs of being affected. Despite having a great capacity to chemisorb this heavy metal, the active sites of the biomass remained intact, retaining around 8.5% of Cr (VI) (as per the table) in the sample. In Elution 2, the amount of Cr (VI) is not as evident as in the other two figures, as the sample only contained around 6.3% of this heavy metal. The adsorption capacity was 8 mg/g, as determined by the isotherm and adsorption kinetics. The chemical elution processes showed that this agent greatly affects the biomass, allowing for its reuse. While the agent does have an impact on the biochemical structure, its predominant effects are seen in the elution and reuse. The images demonstrate that the biomass has a high adsorption capacity and can be chemically eluted and reused to increase this capacity. This means that the biomass can continue to be used for treatment processes without any issues.

### 3.6. Desorption Mechanisms

**Figure 13:**
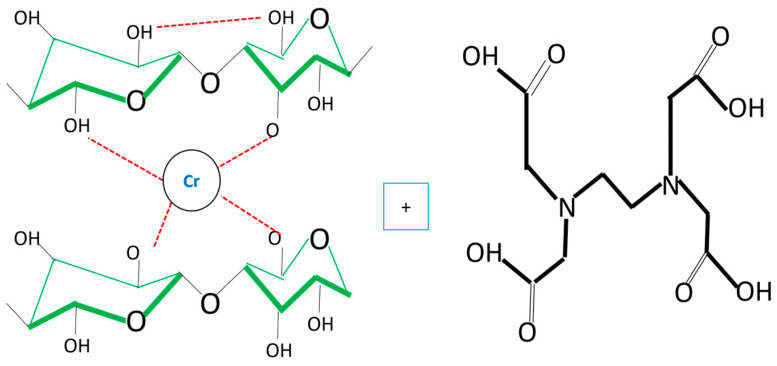
Desorption mechanisms.

The elution mechanism of Cr (VI) by EDTA is proposed based on the experimental and instrumental analyses presented above; this is illustrated in Figure 13.

**Figure 14:**
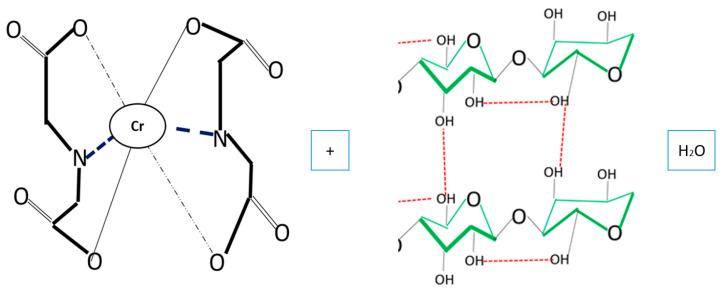
Result of desorption mechanisms.

When EDTA comes into contact with bacterial cellulose biomass with Cr (VI), its hydroxyl groups are protonated [64,65,66,67]; this process completes the elution process. Figure 14 shows the result of the mechanisms of desorption.

The composition of Cr (VI) in the biomass and its elution with EDTA result in the wear of the bacterial cellulose biomass by the chelating agent. However, this biomass is available for another cycle of adsorption and subsequent elution.

## 4. Conclusions

The experiments and the summation of the adsorption capacities demonstrated that bacterial cellulose exhibits a high degree of selectivity in removal processes due to its extensive number of active sites for the chemical adsorption of heavy metals, particularly Cr (VI), and its high resilience capacity in elution processes, rendering it a distinctive biomass for the construction of larger-scale wastewater treatment systems.

The adsorption capacity of the biomass demonstrated a notable increase, reaching 225 mg/g, following four cycles of continuous elution and reuse. This figure represents the cumulative adsorption capacity. This parameter is of great importance in scientific research, particularly in the development of a pilot-scale prototype. The FTIR and SEM spectra facilitated comprehension of the elution and reuse phenomenon of bacterial biomass in the removal of Cr (VI) through EDTA. It is crucial to consider the behavior and suitability of the Langmuir adsorption isotherms. Following each elution, the fit decreased, yet the homogeneity of the adsorption monolayer was maintained. The kinetic adsorption studies on different types of bacterial cellulose prepared for Cr (VI) removal followed the pseudo-second-order model. Furthermore, the simplicity of the production process, the excellent reusability of this material, and its low production cost suggest that this adsorbent could be used in a step-by-step process for the construction of an industrial-scale treatment system.

## Figures and Tables

**Table 1:** Models of isotherm and kinetics.

		Model Isotherm	
Freundlich equation	(1)	$q_{e}=K_{F}{\left(C_{e}\right)}^{n}$	*qe* (mg/g) is the adsorption capacity at equilibrium; (mg/L) is the equilibrium concentration of adsorbents in solution; $K_{f}$ (mg/g) (L/mg) and n are constants for Freundlich [29].
Langmuir equation	(2)	$q_{e}$ = $\frac{q_{m}{K_{l}C}_{e}}{1+K_{l}C_{e}}$	$q_{e}$ (mg/g) is the adsorption capacity at equilibrium; $q_{m}$ (mg/g) is the maximum adsorption capacity; $K_{l}$ (mg/g) is a constant for Langmuir [25].
Sheindorf– Rebuhn–Sheintuch equation (SIPS equation)	(3)	$q_{e}$ = $\frac{q_{m}{\left(k_{e\;}C_{e}\right)}^{1/Ms}}{1+{\left(k_{e\;}C_{e}\right)}^{1/Ms}}$	$q_{e}$ (mg/g) is the adsorption capacity at equilibrium; *qm* (mg/g) is the maximum adsorption capacity; M_s_ is a constant of SIPS [30].
		**Model Kinetic**	
Pseudo-first order	(4)	$q_{t}=q_{e}\left[1-e^{-kt}\right]$	q_t_ and q_e_ (mg/g) are the uptake amounts of pollution at equilibrium and time t (h); K_1_ (min^−1^) is the adsorption rate constant of the pseudo-first order [25,30].
Pseudo-second order	(5)	$q_{t}=\frac{{q_{e}}^{2}Kt}{k\left(q_{e}\right)t+1}$	q_t_ and q_e_ (mg/g) are the uptake amounts of pollution at equilibrium and time t (h); K_2_ is a constant of the second-order model [25].
Intraparticle diffusion	(6)	$q_{t}=K_{id}t^{0.5}+C$	qt (mg/g) is the uptake amount of pollution at equilibrium and time t (h); $K_{id}$ (mg/g)h^0.5^) is the intraparticle diffusion; C (mg/g) is the thickness of the boundary layer [25,30].

**Table 2:** Results of the analysis of the relationship between densities.

Biomass	Mass (g)	Volume Mass (vBc) (mL)	Density Mas (pCb) g/mL	Mass of Particle (mg)	Volume Particle (mm)	Density of Particle (pp)	$\varepsilon =1-\frac{p(Cb)}{pp}$
BC (0)	0.3	0.48	0.62	0.01	0.005	2	0.68
BC (1)	0.3	0.51	0.58	0.01	0.0066	1.5	0.61
BC (2)	0.3	0.54	0.55	0.01	0.007	1.29	0.57
BC (3)	0.3	0.57	0.52	0.01	0.008	1.17	0.55

**Table 3:** Representative isotherms.

	Isotherm	Constant	R^2^
BC	Langmuir	$K_{l}$ = 0.03; q_m_; 75	0.99
	Freundlich	$K_{f}$ = 0.16	0.91
	SIPS	Ms = 0.99	0.97
	**Isotherm**	**Constant**	**R^2^**
BC (1)	Langmuir	$K_{l}$ = 0.02; q_m_; 60	0.99
	Freundlich	$K_{f}$ = 0.11	0.92
	SIPS	M_s_ = 0.99	0.95
	**Isotherm**	**Constant**	**R^2^**
BC (2)	Langmuir	$K_{l}$ = 0.011; q_m_; 55	0.91
	Freundlich	$K_{f}$ = 0.10	0.92
	SIPS	M_s_ = 0.55	0.95
	**Isotherm**	**Constant**	**R^2^**
BC (3)	Langmuir	$K_{l}$ = 0.01; q_m_; 35	0.91
	Freundlich	$K_{f}$ = 0.09	0.98
	SIPS	M_s_ = 0.01	0.91

**Table 4:** Parameters of model kinetics.

	Pseudo-First Order			Pseudo-Second Order			Intraparticle Diffusion		
Samples	q_e_ (mg/g)	K_1_ (min)	R^2^	q_e_ (mg/g)	K_2_ × 10^−3^ (g/mg × min)	R^2^	C (mg/g)	K_d_ (mg/g × 0.5 h)	R^2^
BC(0)	66	0.038	0.94	75	1.4	0.99	18.3	4.4	0.90
BC(1)	55	0.040	0.96	60	1.5	0.98	18.3	4.4	0.93
BC(2)	41	0.042	0.97	55	1.6	0.96	19.2	4.5	0.96
BC(3)	29	0.044	0.99	35	1.8	0.90	20.1	4.6	0.99

**Table 5:** Element representative percentages.

Element	BC	BC (0)%	BC (1)%	BC (2)%	BC (3)%
Carbon	46.8	44.67	46.67	47.67	48.67
Oxygen	48.2	46.94	45.94	47.3	48.3
Cr (VI)	0	13.3	8.5	6.3	2.9

## Data Availability

The original contributions presented in the study are included in the article, further inquiries can be directed to the corresponding author.

## References

[1] Sayago U.F.C., Gómez-Caicedo M.I., Mercado Suárez Á.L. (2024). Design of a sustainable system for wastewater treatment and generation of biofuels based on the biomass of the aquatic plant Eichhornia Crassipes. Sci. Rep..

[2] Sari N.H., Rangappa S.S.M., Siengchin S. (2023). A review on cellulose fibers from Eichornia crassipes: Synthesis, modification, properties and their composites. J. Nat. Fibers.

[3] Sayago U.F.C. (2024). The design of a sustainable industrial wastewater treatment system and the generation of biohydrogen from *E. crassipes*. Polymers.

[4] Carreño Sayago U.F., Castro Y.P., Rivera L.R.C. (2022). Design of a fixed-bed column with vegetal biomass and its recycling for Cr (VI) treatment. Recycling.

[5] Sayago U.F.C., Castro Y.P., Rivera L.R.C., Mariaca A.G. (2020). Estimation of equilibrium times and maximum capacity of adsorption of heavy metals by *E. crassipes*. Environ. Monit. Assess..

[6] Yang H.R., Li S.S., Shan X.C., Yang C., An Q.D., Zhai S.R., Xiao Z.Y. (2021). Hollow polyethyleneimine/carboxymethyl cellulose beads with abundant and accessible sorption sites for ultra-efficient chromium (VI) and phosphate removal. Sep. Purif. Technol..

[7] Qing Q., Shi X.Y., Hu S.Z., Li L., Huang T., Zhang N., Wang Y. (2023). Synchronously Enhanced Removal Ability and Stability of MXene through Biomimetic Modification. Langmuir.

[8] Li L., Shi X.Y., Huang T., Zhang N., Wang Y. (2023). Synchronously enhanced storage stability and adsorption ability of MXene achieved by grafting polyethylenimine. J. Mater. Chem. A.

[9] Sayago U.F.C., Castro Y.P. (2022). Development of a composite material between bacterial cellulose and E crassipes, for the treatment of water contaminated by chromium (VI). Int. J. Environ. Sci. Technol..

[10] Choi S.M., Rao K.M., Zo S.M., Shin E.J., Han S.S. (2022). Bacterial cellulose and its applications. Polymers.

[11] Swingler S., Gupta A., Gibson H., Kowalczuk M., Heaselgrave W., Radecka I. (2021). Recent advances and applications of bacterial cellulose in biomedicine. Polymers.

[12] Parte F.G.B., Santoso S.P., Chou C.C., Verma V., Wang H.T., Ismadji S., Cheng K.C. (2020). Current progress on the production, modification, and applications of bacterial cellulose. Crit. Rev. Biotechnol..

[13] Fernandes I.D.A.A., Pedro A.C., Ribeiro V.R., Bortolini D.G., Ozaki M.S.C., Maciel G.M., Haminiuk C.W.I. (2020). Bacterial cellulose: From production optimization to new applications. Int. J. Biol. Macromol..

[14] Zhong C. (2020). Industrial-scale production and applications of bacterial cellulose. Front. Bioeng. Biotechnol..

[15] Sayago U.F.C. (2021). Design and development of a biotreatment of E. crassipes for the decontamination of water with Chromium (VI). Sci. Rep..

[16] Sayago U.F.C. (2023). Design and Development of a Pilot-Scale Industrial Wastewater Treatment System with Plant Biomass and EDTA. Water.

[17] Gregory D.A., Tripathi L., Fricker A.T., Asare E., Orlando I., Raghavendran V., Roy I. (2021). Bacterial cellulose: A smart biomaterial with diverse applications. Mater. Sci. Eng. R Rep..

[18] Muiruri J.K., Yeo J.C.C., Zhu Q., Ye E., Loh X.J., Li Z. (2023). Bacterial cellulose: Recent advances in biosynthesis, functionalization strategies and emerging applications. Eur. Polym. J..

[19] Peiravi-Rivash O., Mashreghi M., Baigenzhenov O., Hosseini-Bandegharaei A. (2023). Producing bacterial nano-cellulose and keratin from wastes to synthesize keratin/cellulose nanobiocomposite for removal of dyes and heavy metal ions from waters and wastewaters. Colloids Surf. A Physicochem. Eng. Asp..

[20] Chen X., Cui J., Xu X., Sun B., Zhang L., Dong W., Chen C., Sun D. (2020). Bacterial cellulose/attapulgite magnetic composites as an efficient adsorbent for heavy metal ions and dye treatment. Carbohydr. Polym..

[21] Li L., Hu S.Z., Huang T., Zhang N., Wang Y. (2023). Fabricating the ternary CeO_2_@ CNTs/CdSe composite with synchronously enhanced adsorption and photocatalytic activity toward water-soluble pollutants removal. Chem. Eng. J..

[22] Volova T.G., Prudnikova S.V., Kiselev E.G., Nemtsev I.V., Vasiliev A.D., Kuzmin A.P., Shishatskaya E.I. (2022). Bacterial cellulose (BC) and BC composites: Production and properties. Nanomaterials.

[23] Choi S.M., Shin E.J. (2020). The nanofication and functionalization of bacterial cellulose and its applications. Nanomaterials.

[24] Pang M., Huang Y., Meng F., Zhuang Y., Liu H., Du M., Ma Q., Wang Q., Chen Z., Chen L. (2020). Application of bacterial cellulose in skin and bone tissue engineering. Eur. Polym. J..

[25] Li H., Wang Y., Ye M., Zhang X., Zhang H., Wang G., Zhang Y. (2021). Hierarchically porous poly (amidoxime)/bacterial cellulose composite aerogel for highly efficient scavenging of heavy metals. J. Colloid Interface Sci..

[26] Xiaorui K., Cong Z., Pin X., Zhanwen D., Zhijiang C. (2022). Copper ion-imprinted bacterial cellulose for selectively removing heavy metal ions from aqueous solution. Cellulose.

[27] Kaur J., Sengupta P., Mukhopadhyay S. (2022). Critical review of bioadsorption on modified cellulose and removal of divalent heavy metals (Cd, Pb, and Cu). Ind. Eng. Chem. Res..

[28] Cheng R., Kang M., Zhuang S., Shi L., Zheng X., Wang J. (2019). Adsorption of Sr (II) from water by mercerized bacterial cellulose membrane modified with EDTA. J. Hazard. Mater..

[29] Guo D.M., An Q.D., Xiao Z.Y., Zhai S.R., Shi Z. (2017). Polyethylenimine-functionalized cellulose aerogel beads for efficient dynamic removal of chromium (VI) from aqueous solution. RSC Adv..

[30] Yang H.R., Li S.S., Yang C., An Q.D., Zhai S.R., Xiao Z.Y. (2022). Bi-layered hollow amphoteric composites: Rational construction and ultra-efficient sorption performance for anionic Cr (VI) and cationic Cu (II) ions. J. Colloid Interface Sci..

[31] Luo H., Feng F., Yao F., Zhu Y., Yang Z., Wan Y. (2020). Improved removal of toxic metal ions by incorporating graphene oxide into bacterial cellulose. J. Nanosci. Nanotechnol..

[32] Sayago U.F.C., Ballesteros V.A.B. (2024). The Design of a Process for Adsorbing and Eluting Chromium (VI) Using Fixed-Bed Columns of *E. crassipes* with Sodium Tripolyphosphate (TPP). Water.

[33] Ding R., Hu S., Xu M., Hu Q., Jiang S., Xu K., Tremblay P.L., Zhang T. (2021). The facile and controllable synthesis of a bacterial cellulose/polyhydroxybutyrate composite by co-culturing Gluconacetobacter xylinus and Ralstonia eutropha. Carbohydr. Polym..

[34] Park D., Yun Y.S., Park J.M. (2006). Mechanisms of the removal of hexavalent chromium by biomaterials or biomaterial-based activated carbons. J. Hazard. Mater..

[35] Farag S., Ibrahim H.M., Amr A., Asker M.S., El-Shafie A. (2019). Preparation and characterization of ion exchanger based on bacterial cellulose for heavy metal cation removal. Egypt. J. Chem..

[36] Tohamy H.A.S. (2024). Fluorescence ‘Turn-on’Probe for Chromium Reduction, Adsorption and Detection Based on Cellulosic Nitrogen-Doped Carbon Quantum Dots Hydrogels. Gels.

[37] Li D., Tian X., Wang Z., Guan Z., Li X., Qiao H., Ke H., Luo L., Wei Q., Huang J. (2020). Multifunctional adsorbent based on metal-organic framework modified bacterial cellulose/chitosan composite aerogel for high efficient removal of heavy metal ion and organic pollutant. Chem. Eng. J..

[38] Hashem A., Taha G.M., Fletcher A.J., Mohamed L.A., Samaha S.H. (2021). Highly efficient adsorption of Cd (II) onto carboxylated camelthorn biomass: Applicability of three-parameter isotherm models, kinetics, and mechanism. J. Polym. Environ..

[39] Sayago U.F.C., Ballesteros V.B. (2023). Recent advances in the treatment of industrial wastewater from different celluloses in continuous systems. Polymers.

[40] Gu S., Lan C.Q. (2024). Mechanism of heavy metal ion biosorption by microalgal cells: A mathematic approach. J. Hazard. Mater..

[41] Yu X., Tong S., Ge M., Wu L., Zuo J., Cao C., Song W. (2013). Adsorption of heavy metal ions from aqueous solution by carboxylated cellulose nanocrystals. J. Environ. Sci..

[42] Lu J., Jin R.N., Liu C., Wang Y.F., Ouyang X.K. (2016). Magnetic carboxylated cellulose nanocrystals as adsorbent for the removal of Pb (II) from aqueous solution. Int. J. Biol. Macromol..

[43] Tran H.N., You S.J., Hosseini-Bandegharaei A., Chao H.P. (2017). Mistakes and inconsistencies regarding adsorption of contaminants from aqueous solutions: A critical review. Water Res..

[44] Chen Y., Long Y., Li Q., Chen X., Xu X. (2019). Synthesis of high-performance sodium carboxymethyl cellulose-based adsorbent for effective removal of methylene blue and Pb (II). Int. J. Biol. Macromol..

[45] Phaenark C., Nasuansujit S., Somprasong N., Sawangproh W. (2024). Moss biomass as effective biosorbents for heavy metals in contaminated water. Heliyon.

[46] Freundlich H.M.F. (1906). Over the adsorption in solution. J. Phys. Chem.

[47] Amar M.B., Mallek M., Valverde A., Monclús H., Myers T.G., Salvadó V., Cabrera-Codony A. (2024). Competitive heavy metal adsorption on pinecone shells: Mathematical modelling of fixed-bed column and surface interaction insights. Sci. Total Environ..

[48] Chao H.P., Chang C.C., Nieva A. (2014). Biosorption of heavy metals on Citrus maxima peel, passion fruit shell, and sugarcane bagasse in a fixed-bed column. J. Ind. Eng. Chem..

[49] Safardastgerdi M., Ardejani F.D., Mahmoodi N.M. (2023). Lignocellulosic biomass functionalized with EDTA dianhydride for removing Cu (II) and dye from wastewater: Batch and fixed-bed column adsorption. Miner. Eng..

[50] Solgi M., Mohamed M.H., Udoetok I.A., Steiger B.G., Wilson L.D. (2024). Evaluation of a granular Cu-modified chitosan biocomposite for sustainable sulfate removal from aqueous media: A batch and fixed-bed column study. Int. J. Biol. Macromol..

[51] Lu S., Liu W., Wang Y., Zhang Y., Li P., Jiang D., Fang C., Li Y. (2019). An adsorbent based on humic acid and carboxymethyl cellulose for efficient dye removal from aqueous solution. Int. J. Biol. Macromol..

[52] Lin Y.C., Ho S.H., Zhou Y., Ren N.Q. (2018). Highly efficient adsorption of dyes by biochar derived from pigments-extracted macroalgae pyrolyzed at different temperature. Bioresour. Technol..

[53] Liu J., Chen T.-W., Yang Y.-L., Bai Z.-C., Xia L.-R., Wang M., Lv X.-L., Li L. (2020). Removal of heavy metal ions and anionic dyes from aqueous solutions using amide-functionalized cellulose-based adsorbents. Carbohydr. Polym..

[54] Yuvaraja G., Pang Y., Chen D.-Y., Kong L.-J., Mehmood S., Subbaiah M.V., Rao D.S., Pavuluri C.M., Wen J.-C., Reddy G.M. (2019). Modification of chitosan macromolecule and its mechanism for the removal of Pb (II) ions from aqueous environment. Int. J. Biol. Macromol..

[55] Li X., Qi Y., Li Y., Zhang Y., He X., Wang Y. (2013). Novel magnetic beads based on sodium alginate gel crosslinked by zirconium (IV) and their effective removal for Pb2+ in aqueous solutions by using a batch and continuous systems. Bioresour. Technol..

[56] Sayago U.F.C., Ballesteros V.B. (2023). Development of a treatment for water contaminated with Cr (VI) using cellulose xanthogenate from E. crassipes on a pilot scale. Sci. Rep..

[57] Cao J., Tan Y., Che Y., Xin H. (2010). Novel complex gel beads composed of hydrolyzed polyacrylamide and chitosan: An effective adsorbent for the removal of heavy metal from aqueous solution. Bioresour. Technol..

[58] Oyewo O.A., Elemike E.E., Onwudiwe D.C., Onyango M.S. (2020). Metal oxide-cellulose nanocomposites for the removal of toxic metals and dyes from wastewater. Int. J. Biol. Macromol..

[59] Tohamy H.A.S., El-Sakhawy M., Kamel S. (2023). Microwave-assisted synthesis of amphoteric fluorescence carbon quantum dots and their chromium adsorption from aqueous solution. Sci. Rep..

[60] Alipour A., Zarinabadi S., Azimi A., Mirzaei M. (2020). Adsorptive removal of Pb (II) ions from aqueous solutions by thiourea-functionalized magnetic ZnO/nanocellulose composite: Optimization by response surface methodology (RSM). Int. J. Biol. Macromol..

[61] Qiao H., Zhou Y., Yu F., Wang E., Min Y., Huang Q., Pang L., Ma T. (2015). Effective removal of cationic dyes using carboxylate-functionalized cellulose nanocrystals. Chemosphere.

[62] Putro J.N., Santoso S.P., Soetaredjo F.E., Ismadji S., Ju Y.H. (2019). Nanocrystalline cellulose from waste paper: Adsorbent for azo dyes removal. Environ. Nanotechnol. Monit. Manag..

[63] Oyewo O.A., Mutesse B., Leswifi T.Y., Onyango M.S. (2019). Highly efficient removal of nickel and cadmium from water using sawdust-derived cellulose nanocrystals. J. Environ. Chem. Eng..

[64] Villalobos-Rodríguez R., Montero-Cabrera M.E., Esparza-Ponce H.E., Herrera-Peraza E.F., Ballinas-Casarrubias M.L. (2012). Uranium removal from water using cellulose triacetate membranes added with activated carbon. Appl. Radiat. Isot..

[65] Mao N., Yang L., Zhao G., Li X., Li Y. (2012). Adsorption performance and mechanism of Cr (VI) using magnetic PS-EDTA resin from micro-polluted waters. Chem. Eng. J..

[66] Novotnik B., Ščančar J., Milačič R., Filipič M., Žegura B. (2016). Cytotoxic and genotoxic potential of Cr (VI), Cr (III)-nitrate and Cr (III)-EDTA complex in human hepatoma (HepG2) cells. Chemosphere.

[67] Sayago U.F.C., Ballesteros V.B., Aguilar A.M.L. (2024). Designing, Modeling and Developing Scale Models for the Treatment of Water Contaminated with Cr (VI) through Bacterial Cellulose Biomass. Water.

